# Sharing the sandbox: Evolutionary mechanisms that maintain bacterial cooperation

**DOI:** 10.12688/f1000research.7363.1

**Published:** 2015-12-23

**Authors:** Eric Bruger, Christopher Waters

**Affiliations:** 1 Department of Microbiology and Molecular Genetics and the BEACON Center for the Study of Evolution in Action, Michigan State University, East Lansing, MI, 48824, USA

**Keywords:** microbes, biofilm, bacterial cooperation

## Abstract

Microbes are now known to participate in an extensive repertoire of cooperative behaviors such as biofilm formation, production of extracellular public-goods, group motility, and higher-ordered multicellular structures. A fundamental question is how these cooperative tasks are maintained in the face of non-cooperating defector cells. Recently, a number of molecular mechanisms including facultative participation, spatial sorting, and policing have been discovered to stabilize cooperation. Often these different mechanisms work in concert to reinforce cooperation. In this review, we describe bacterial cooperation and the current understanding of the molecular mechanisms that maintain it.

## Introduction

Bacteria were once thought to be solitary individuals, but it is now clear that they lead complex social lives ^1, 2^. Multicellular bacterial communities termed biofilms are now considered a normal form of bacterial growth. Bacterial chemical communication, including quorum sensing (QS), is ubiquitous ^3, 4^, and the molecular underpinnings of multicellular bacterial structures such as *Myxococcus xanthus* fruiting bodies and *Streptomyces* filaments are also being elucidated ^5, 6^. With our increased understanding of bacterial sociality comes a further appreciation of the role of cooperation in many bacterial processes. Microbial cooperative behaviors have important impacts on our own lives, including antibiotic resistance ^7^, biofilm formation in chronic infections ^8^, and virulence during acute infections ^9, 10^. Explaining the evolution of cooperative tasks has long challenged evolutionary biology, as these systems appear ripe for exploitation by non-cooperating defector/cheater cells that receive the benefits of cooperation without paying the cost of production ^11^. Because of their short generation times, large population sizes, small genomes, and asexual reproduction, bacteria are now recognized as ideal model systems to understand the factors leading to the evolution and persistence of cooperative behaviors ^12– 14^. In this review, we will summarize from both a conceptual and a mechanistic perspective our understanding of how cooperation is maintained in bacteria.

## Facultative cooperation

Bacteria have evolved complex regulatory circuitry to respond and effectively acclimate to different environments, so it is not surprising that this flexible regulatory circuitry can also be utilized to control cooperative traits. Cooperative behaviors in bacteria, such as the production of extracellular “public good” molecules, defined as resources that can be utilized by both the producers and the non-producers in the community, are exploitable by non-producing cheater/defector cells. One approach to limit cheater invasion is facultative cooperation. Engaging in cooperation at limited times, particularly when the benefit is the greatest, or in environmental conditions where the cost of cooperation is low can limit or prevent cheater invasion ^15, 16^. In this way, bacteria may preserve cooperation in conditions that would otherwise favor its collapse ^17^. It is notable, however, that facultative participation only partly mediates the problem of cooperation by limiting the times when a cell must maintain it. Other mechanisms, such as relatedness, are likely required in conjunction with optional participation to preserve cooperation.

**Figure 1.**
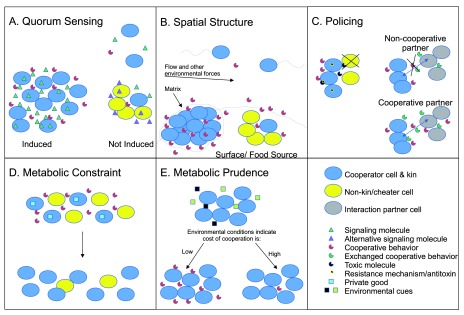
Mechanisms that act to maintain cooperation. **A.** **Quorum Sensing**. The cooperative behavior is induced only when a sufficient amount of signal has accumulated (left). **B.** **Spatial Structure**. When cells are able to assort with kin in space, particularly in the case of biofilm formation (bottom left), dispersal of cells and diffusion of public goods are limited and promote the maintenance of cooperative behavior. **C.** **Policing**. This mechanism may act through directed harm (left) or restraint of benefits (right). **D.** **Metabolic Constraint.** Producers of a cooperative behavior such as a public good also produce an individually retained private good that is beneficial or required for survival and growth in the focal environment. **E.** **Metabolic Prudence.** Cells detect nutrients and other cues in their environment to determine whether it is cost effective to cooperate.

 For public goods to be effective, they often must exceed a threshold concentration in the extracellular environment ^15^. Therefore, there must be a sufficient number of producing cells contributing to the public good. For this reason, production of many public goods such as exoenzymes, proteases, chitinases, and siderophores are regulated by QS ( Figure 1A) ^4^. This process relies on the secretion and detection by bacteria of small chemical signals known as autoinducers into the extracellular environment. As the cell density of a growing culture increases, so does the concentration of autoinducers. This is reinforced by the positive feedback of many QS system on autoinducer synthesis ^18, 19^. At a specific concentration of signal, receptors bind to and sense these autoinducers, allowing the bacteria to switch from a low- to high-cell density state. This is often seen as a transition from non-production to production of cooperative traits such as extracellular public goods. QS itself is an exploitable cooperative behavior, as QS-specific cheaters that do not signal, overproduce signal, or do not respond to signal can evolve ^20^.

QS regulation of cooperation has been well studied in the bacterium *Pseudomonas aeruginosa*. This bacterium upregulates extracellular proteases in the high-cell density state ^21^. These proteases degrade extracellular proteins, liberating smaller peptides that can be used for growth. Thus, the growth of *P. aeruginosa* in minimal media with the casein protein family as a carbon source is dependent upon a functional QS system. Mutants in the QS pathway do not secrete high levels of proteases and grow poorly in this environment, but they receive negative frequency-dependent fitness benefits when mixed with a cooperating strain ^20^. In other words, QS mutants can invade the wild-type strain when rare but lose their fitness benefits when common, a feature likely to occur in most public goods scenarios ^20, 22, 23^. Therefore, although QS limits maximum public good production to high-cell density, it is not sufficient in this case to completely prevent cheater invasion, although it may mediate the extent to which this occurs. Similar results in a casein growth medium were recently described for *Vibrio cholerae* ^24^ and *Vibrio harveyi* (unpublished results from our laboratory) *,* which also secrete proteases at high-cell density in a QS-dependent manner. The degree of resistance to cheating QS can provide likely depends upon the cost and benefit functions of the behavior, the manner in which regulation is imposed, and the genetic architecture of the QS system.

As QS itself does not appear sufficient to completely ward off cheaters, at least in these systems, additional mechanisms for cheater prevention are required. One such mechanism, referred to as metabolic constraint, found that the utilization of adenosine was also positively controlled by QS ^24^ ( Figure 1D). Unlike protease secretion, which is a public good, adenosine utilization primarily benefits the producing individual, making this function as a “private good”. The addition of adenosine to the medium inhibited the evolution of QS mutants, as these were not able to effectively use this resource. Therefore, the cooperators were able to access a benefit that was unavailable to the defectors. It was proposed that mutually regulating public and private goods under control of QS could be a mechanism to stabilize QS-controlled cooperative tasks. However, if utilization of adenosine is a private good that benefits the producer in a density-independent manner, it is not clear how this system would have evolved. An alternative hypothesis is that in its natural environment adenosine is primarily present in high-cell density situations. QS could function as an environmental cue to prime *P. aeruginosa* to utilize likely nutrient sources that may be encountered at different cell densities. While QS can explain varying behavioral differences with density, the underlying cooperative behaviors are still promoted when cells have unified interests, such as relatedness. Because of autoinducer specificity, QS provides not just evidence of general density but also the degree of relatedness of the surrounding community. It is therefore our view that metabolic constraint systems will only evolve when utilization of the private good in a density-dependent manner is favored. On a broader note, any condition in which co-regulation of the public and private good is most beneficial could exhibit metabolic constraint.

Another mechanism to limit cheater invasion is to only produce public goods when their cost is minimal, an idea termed metabolic prudence ( Figure 1E). *P. aeruginosa* also produces carbon-rich molecules called rhamnolipids that allow large numbers of these organisms to “swarm” over certain surfaces, such as soft agar plates. Rhamnolipids are a public good that can be exploited by non-producers to swarm. Xavier and colleagues noticed ^25^, however, that a non-producer did not exhibit higher fitness than a rhamnolipid producer during a swarm assay of chimeric populations, even though a significant portion of carbon was being directed towards synthesis of this public good. The authors deduced that *P. aeruginosa* only produced rhamnolipids when experiencing an excess of carbon in relation to nitrogen levels. Thus, the cost of production for this public good was minimized and did not lead to reduced fitness versus the non-producer. Interestingly, rhamnolipid production is also regulated by QS, indicating that production only occurs at high density and/or relatedness as well ^26^.

Metabolic prudence is thus a facultative cooperation mechanism, which illustrates that bacteria integrate the relative cost associated with cooperative traits. Though it has not yet been widely demonstrated, it is likely to occur for additional microbial cooperative behaviors. It is common for complex traits to be controlled by multiple regulatory inputs. For example, the catabolite repressor protein (CRP), which responds to the presence of phosphotransfer sugars, at least in *Escherichia coli,* regulates 300 genes, which is ~7% of its genome ^27^. It is our opinion that regulatory connections between QS (or other signaling systems), central metabolism, and the control of cooperation will be common, and finding other systems that demonstrate metabolic prudence will be an exciting new avenue of research ^28^.

## Spatial structure and assortment

Spatial structuring of related cooperators is a key mechanism by which cooperation is likely evolved and maintained ^11, 29, 30^ ( Figure 1B). One critical example of cells actively structuring themselves in an environment that is proposed to encourage cooperation is the production of biofilms ^31^. Biofilms are multicellular communities of bacteria encased in an extracellular matrix. A costly and potentially cheatable behavior itself ^32^, biofilm formation provides a framework for cells to situate themselves in space and direct cooperative benefits preferentially towards clonal offspring and other related kin. Biofilms also restrict diffusion so that public goods, such as extracellular enzymes, remain near the producing cell rather than being dispersed by flow or other forces ^33, 34^. This was recently demonstrated as *V. cholerae* biofilms attached to chitin surfaces retain sufficient amounts of the extracellular enzymes chitinases to metabolize this nutrient ^33^. Cells that do not form biofilms lose higher portions of these public goods due to increased loss via diffusive and advective forces, which likely reflects conditions encountered in natural environments ^33, 34^. This means that public goods benefits remain distributed over a narrower, more local range in space that favors their diversion toward neighboring kin cells. Additionally, biofilm formers may even be able to exclude non-producers from colonized nutrient source surfaces ^35^.

However, this structuring also comes with the potential for more competition between kin, especially if cells don't also possess the capacity to disperse and colonize new patches in their habitat. For example, experimentally evolved lineages that produce more biofilm through an evolved wrinkly spreader lifestyle are able to bind tightly to neighboring cells due to enhanced production of extracellular matrix materials, but this typically comes with a tradeoff for growth potential ^36, 37^. The tradeoff may be in part restrained by the reduced ability of cells to disperse from a cluster. A similar example of a tradeoff between colonization and dispersal is seen in *V. cholerae*, where biofilm producers compete and grow better on a surface but are less effective at dispersing to new locations in their habitat ^38^, and natural populations of *Vibrio cyclitrophicus* demonstrate differential specialization for colonization onto and dispersal from particles, signifying that this phenomenon could be more widespread ^39^. This suggests that multiple selective pressures are naturally acting upon microbes that can either reinforce or act against other cooperative behaviors.

In the examples described thus far, no assumptions have been made about the ability of cells to recognize the presence or identity of neighboring cells, and this is not always theoretically necessary for spatial structure to enable cooperation ^40, 41^. However, it may be important to be able to recognize neighbors and restrain competition if surrounded primarily by relatives. There are many examples of cells being capable of effectively distinguishing between self and non-self and adjusting behavior accordingly in ways that impact growth outcomes ^42– 44^. QS is one such system in bacteria, but other contact-dependent recognition systems such as the contact-dependent growth inhibition (CDI) system of *Burkholderia*, type VI secretion systems, and flocculation in yeast have been described ^45– 47^. This ability to correctly decipher amongst neighbors and the composition of the surrounding community could greatly encourage the success of cooperation, particularly if production of cooperative behaviors is predicated upon sensing members of a cell's own genotype.

It is worth noting that producer cells at low frequency can in some cases preferentially gain the benefit of public goods production compared with non-producers, even in the absence of higher ordered structure ^48^. This creates a snowdrift scenario whereby the rare type has an advantage. This situation is, however, highly dependent on the parameters of the specific cooperative behavior, but it may contribute to maintenance of cooperative tasks.

## Policing

Policing and related forms of punishment are proposed as another mechanism to stabilize cooperation in the face of potential cheaters ( Figure 1C). In this scenario, an aggressive action that negatively impacts fitness targets cheaters relative to cooperators ^49^. Policing has been commonly observed among many eukaryotic organisms ^50^, including insect workers, birds, and social primates, but the prevalence and diversity of molecular mechanisms underlying bacterial policing are not well characterized. Punishment could be enacted by either restraining benefits directed to a non-contributing partner or by direct harm. In the second case, this behavior may be costly to the enacting individual but still stabilize other cooperative behaviors for cooperating kin in the population as long as the punishment and resulting cooperation are positively correlated ^51, 52^.

One example of policing is the enforcement of sanctions, seen in the interaction of the root-nodule forming bacteria *Rhizobia* with its host plant *.* Symbionts that do not sufficiently contribute fixed nitrogen to their associated host receive a limited flow of oxygen and nutrients in return ^53, 54^. Limiting benefits or imposing costs to less or non-cooperative partners in this manner should favor more cooperative partners in mutualisms ^54, 55^. In some systems, such as squid- *Vibrio* symbioses, the host is able to filter and selectively favor suitable partners in such a manner, and host-enforced bottlenecks are also likely to play a strong role in maintaining fidelity in the interaction ^56, 57^. Bottlenecks may more generally act to stabilize cooperative behavior, regardless of host association ^58– 60^. In this way, restraining a benefit in the face of non-reciprocating partners can have the effect of maintaining the interaction. These sanctions need not be restricted to inter-species interactions and could be imagined to occur, for instance, in populations where cells are exchanging metabolites ^61^.

Recently, QS in *P. aeruginosa* has also been shown to induce policing that targets QS defectors by regulating cyanide production and resistance ^62^. As described above, QS induction of extracellular proteases is necessary for maximum growth in a minimal media environment with casein as the carbon source. In this case, certain classes of QS defectors are unable to produce co-regulated compounds that counteract the effects of cooperator-produced cyanide and are thus unable to completely invade a QS-proficient population cooperating via extracellular enzyme production that could otherwise be exploited by the potential cheats.

In all likelihood, these types of policing mechanisms may be difficult to maintain and unlikely to be common for maintaining bacterial cooperation ^11^. Because policing behaviors are costly to perform and may target related kin, they may convey a fitness disadvantage under many conditions. However, this effect may be somewhat alleviated if such traits are only expressed conditionally, as shown in the QS-regulated example. For cooperative partners, from either the same or different species, sanctions may arise more naturally. Due to negative effects on the productivity caused by a poor partner, reciprocal sanctioning effects will more naturally emerge, as fewer partners will be present to repay the favor ^40^.

## Division of labor in bacteria

A penultimate form of cooperation that is a requirement for the development of higher ordered multicellularity is “division of labor”. Division of labor can be defined as cooperating individuals that perform discrete tasks that are themselves costly to the individuals, but the sum total of this task distribution is beneficial to the larger community. Division of labor is clearly evident in complex multicellular eukaryotes. A heart cell has differentiated to perform very different tasks than a liver cell. In these organisms, development and terminal differentiation are keys to driving and maintaining phenotypic heterogeneity. Division of labor has also clearly been observed in bacteria. A classic example is spore formation by *M. xanthus* ^63^. Upon starvation, this predatory bacterium aggregates into multicellular mounds, which ultimately form structures called fruiting bodies coated with environmentally resistant *M. xanthus* spores that rise above the local surface. These structures are thought to aid in dispersal of the spores to new environments.

 Division of labor is also proposed to be a common feature of biofilms, although this is a controversial idea that as of yet has little experimental support. Indeed, five specific *Bacillus subtilis* cell types can be observed in a monospecies biofilm ^64^. These subtypes localize to distinct regions of the biofilm, but the adaptive function of these cell types is not clear, as a locked matrix-producing cell type is sufficient to produce a robust biofilm in the lab. However, it is likely that a homogenous biofilm would be maladaptive in the natural life cycle of *B. subtilis,* as the laboratory environment is missing key aspects of the natural world. It also seems unlikely that biofilms will remain as genetically homogenous as liquid cultures due to limited dispersal leading to restriction of competition to local scales. Understanding the evolutionary factors that drive the emergence of phenotypic heterogeneity ** must rely on a better understanding of the ecology of these bacteria and ideally guide experiments on these organisms in more naturally relevant systems. Moreover, like all forms of cooperation, identifying the strategies and mechanisms that maintain these interactions in the face of defectors will be an intriguing area of research.

The above examples represent division of labor in monospecies systems, but interspecies division of labor can and does occur as well. In complex microbial communities, we predict that division of labor will be most evident in the cooperation of individuals through metabolic exchanges ^65, 66^. Indeed, this has been observed in clinical cystic fibrosis isolates of *Staphylococcus aureus* ^67^. This phenomenon has been shown to be possible in synthetic as well as natural communities ^40, 68– 70^. In mixed communities, members that are not directly involved in the exchange may still impact it providing a degree of separation or assortment of individual partners, a process labeled social insulation ^40, 71^. With the increasing importance of the human and animal microbiome in health and disease, understanding cooperative division of labor interactions in these communities, and potentially with the host, will be an increasingly important area in microbial evolution.

## Conclusion

As we have seen, microbial cooperation occurs in diverse manners, and the mechanisms guiding its maintenance are likewise diverse. These mechanisms all fundamentally act to mediate the fitness costs imposed by expressing cooperative behavior or by altering the way that benefits are administered. Fitness gains may be directed to the acting party (direct), to related kin (indirect), or both, and thus act to increase the organism's overall inclusive fitness. Also evident throughout this review, these mechanisms may and often do work in concert with one another. As researchers discover novel examples of microbial cooperation, more mechanisms that direct their maintenance will likely come to light. We are particularly optimistic that many mechanisms of cooperation utilizing optional participation guided by communication will be discovered in experimental and natural communities. The surface has only been scratched in this area of research, and it will be exciting to see what new mechanisms are uncovered, how they may potentially be used for industrial and medical applications, as well as how they may inform what we know about biology at both the micro and macro scale.

## Abbreviations

QS, quorum sensing.
